# Does Dietary Mitigation of Enteric Methane Production Affect Rumen Function and Animal Productivity in Dairy Cows?

**DOI:** 10.1371/journal.pone.0140282

**Published:** 2015-10-28

**Authors:** Jolien B. Veneman, Stefan Muetzel, Kenton J. Hart, Catherine L. Faulkner, Jon M. Moorby, Hink B. Perdok, Charles J. Newbold

**Affiliations:** 1 Cargill Innovation Center, Velddriel, The Netherlands; 2 Institute of Biological, Environmental and Rural Sciences, Aberystwyth University, Aberystwyth, United Kingdom; 3 AgResearch Limited, Grasslands Research Centre, Palmerston North, New Zealand; Catalan Institute for Water Research (ICRA), SPAIN

## Abstract

It has been suggested that the rumen microbiome and rumen function might be disrupted if methane production in the rumen is decreased. Furthermore concerns have been voiced that geography and management might influence the underlying microbial population and hence the response of the rumen to mitigation strategies. Here we report the effect of the dietary additives: linseed oil and nitrate on methane emissions, rumen fermentation, and the rumen microbiome in two experiments from New Zealand (Dairy 1) and the UK (Dairy 2). Dairy 1 was a randomized block design with 18 multiparous lactating cows. Dairy 2 was a complete replicated 3 x 3 Latin Square using 6 rumen cannulated, lactating dairy cows. Treatments consisted of a control total mixed ration (TMR), supplementation with linseed oil (4% of feed DM) and supplementation with nitrate (2% of feed DM) in both experiments. Methane emissions were measured in open circuit respiration chambers and rumen samples were analyzed for rumen fermentation parameters and microbial population structure using qPCR and next generation sequencing (NGS). Supplementation with nitrate, but not linseed oil, decreased methane yield (g/kg DMI; P<0.02) and increased hydrogen (P<0.03) emissions in both experiments. Furthermore, the effect of nitrate on gaseous emissions was accompanied by an increased rumen acetate to propionate ratio and consistent changes in the rumen microbial populations including a decreased abundance of the main genus *Prevotella* and a decrease in archaeal mcrA (log_10_ copies/ g rumen DM content). These results demonstrate that methane emissions can be significantly decreased with nitrate supplementation with only minor, but consistent, effects on the rumen microbial population and its function, with no evidence that the response to dietary additives differed due to geography and different underlying microbial populations.

## Introduction

Livestock are estimated to be responsible for 14.5% of the total greenhouse gas (GHG) emission from anthropogenic sources [1], with methane resulting from enteric fermentation the second largest source of anthropogenic GHG, representing 39% of the livestock sector emissions [1]. Numerous studies have investigated the potential to decrease methane from enteric fermentation in ruminants using dietary strategies or dietary additives [2]. However, relatively few studies have considered the wider consequences of these interventions on the functioning of the rumen microbial ecosystem [3]. It has been suggested that rumen function will be disrupted if rumen methane production is inhibited without the provision of alternative hydrogen sinks [4]. Here we report on the effect of two dietary additives (nitrate and linseed oil) selected based on a previous meta-analysis as persistent and potentially practical methane mitigation additives [5] on rumen fermentation, methane and hydrogen emissions and the rumen microbiome. Furthermore, given the concerns that geography and management might influence the underlying microbial population and hence the response to the additive [6], the additives were tested in two matched experiments in New Zealand and in the UK.

## Materials and Methods

### Experimental design

Experiments were conducted at the Ulyatt-Reid Large Animal Facility AgResearch Grasslands Research Centre in Palmerston North (New Zealand) (Dairy 1) and Trawsgoed Research Farm, Aberystwyth University (UK) (Dairy 2). All procedures were approved and regulated by the Animal Ethics Committee of AgResearch Limited or Aberystwyth University’s Animal Welfare and Ethical Review Board (under the regulations of UK Home Office Animals (Scientific Procedures) Act, 1986).

Dairy 1 was a randomized block design experiment, each block lasting up to 40 days in total with 6 blocks of cows (*n* = 18). Cows were adapted to the treatments for at least 14 days (14-39d) before a measurement period of 10 days. Measurement periods were started half a week apart with the last block finishing at 40 days. Eighteen multiparous lactating Holstein (n = 12) and Holstein-Jersey cross (n = 6) dairy cows 180±21 days in milk (DIM), 533±65 kg body weight (BW) and 20.5±2.6 L/d milk yield (mean ± standard deviation) were blocked according to breed, milk yield and BW and randomly assigned to one of three treatments. During the adaptation phase animals were housed by treatment in outdoor concrete pens with wood shavings as bedding. The TMR diet (Table 1) was offered *ad libitum* twice daily directly after each milking. Refusals were removed before the morning feeding. For the measurement period cows were moved to the Ulyatt-Reid Facility and housed in individual pens with concrete flooring and rubber mats. Feed was offered twice daily *ad libitum* to determine feed intake and then restricted to 95% of the DMI of the animal consuming the smallest amount within a block starting at day 5 of the measurement period until the end of the measurement period. Throughout the experiment animals were milked twice daily at 0730 h and 1600 h. Water was available *ad libitum* and BW was measured weekly.

**Table 1:** Ingredient and chemical composition of the control, linseed and nitrate diets offered to lactating dairy cows in Dairy 1 and Dairy 2.

Ingredient, g/kg DM	Dairy 1			Dairy 2		
	Control	Linseed	Nitrate	Control	Linseed	Nitrate
Maize silage	695	648	695	300	300	300
Grass silage	-	-	-	300	300	300
Concentrate^1^	-	-	-	333	333	333
Soy bean meal	169	176	169	-	-	-
Lucerne chaff	98	98	98	-	-	-
Mineral premix	7	7	7	-	-	-
Magnesium oxide	0.7	0.7	0.7	-	-	-
Magnesium sulfate	3.0	3.0	3.0	-	-	-
Rumen inert fat^2^	-	-	-	40	-	40
Linseed oil	-	40	-	-	40	-
Urea	10.8	10.8	-	10.8	10.8	-
Calcinit™^3^	-	-	27.1	-	-	27.1
Limestone	16.4	16.4	-	16.4	16.4	-
Chemical composition						
Dry matter, g/kg product	458	482	462	364	369	364
Gross energy, MJ/kg DM	17.5	18.3	17.9	18.4	18.6	18.2
Crude protein^4^, g/kg DM	175	179	179	154	150	150
Crude fat, g/kg DM	22	62	22	50	65	61
Crude ash, g/kg DM	62	61	60	68	69	70
Starch, g/kg DM	222	208	223	167	180	167
NDF, g/kg DM	350	335	333	424	397	413
ADF, g/kg DM	201	185	185	202	201	201
Calcium, g/kg DM	8.6	8.6	8.6	9.7	9.9	9.6
Phosphorus, g/kg DM	2.6	2.6	2.6	3.1	3.2	3.2
Sulfur, g/kg DM	2.3	2.3	2.2	2.1	2.1	2.1
Nitrate, g/kg DM	0.07	0.07	14.3	<0.2	<0.2	20.4

ADF, acid detergent fiber; DM, dry matter; ND; not determined; NDF neutral detergent fiber.

^1^ Ingredient composition in g/kg DM: barley 511, sugar beet pulp 284, molasses 80, barley distillers grains 50, Sopralin 40 (protected soybean meal, Trouw Nutrition, Wincham, UK), premix 19 (containing in g/kg DM: Ca 0, P 70, Na 70, Mg 250, Mn 2, Cu 3, Zn 4, I 0.8, Se 0.045, Co 0.006; vitamins in mg/kg DM: retinol 165, cholecalciferol 2.75, α-tocopherol 6670, cobalamin 20).

^2^ ButterfatExtra™, Trident, AB Agri ltd., Peterborough, UK.

^3^ Nitrate source (Yara, Oslo, Norway) with molecular formula. 5Ca(NO_3_)_2_∙NH_4_NO_3_∙10H_2_O; 75% NO_3_ in DM.

^4^ N × 6.25.

Dairy 2 was a duplicated 3 x 3 Latin square design experiment with blocks of 3 cows and 32 day periods and blocks starting 7 days apart. Six rumen cannulated Holstein-Friesian lactating dairy cows 182±16 DIM, 578±52 kg BW and 21.5±4.5 L/d milk yield were housed in individual stalls for the entire experiment. Animals were milked twice a day at 0700 h and 1600 h. The TMR diet (Table 1) was offered *ad libitum* at 0800h daily, with refusals removed just before feeding and water was available *ad libitum* throughout the entire experiment. Between each period, animals were fed the control diet for 3 days and allowed to exercise outside in a concrete pen with a roofed shelter and straw bedding.

### Treatments

In Dairy 1, animals were adapted to the control diet for 7 days before the start of the experiment. Diets were prepared fresh before each feeding. In Dairy 2, animals were adapted to the control diet for 9 days prior to the start of the first period of the first square of cows. Diets were prepared on a weekly basis and feed was stored at 4°C until feeding. In both experiments the treatments consisted of a control diet, dietary supplementation of linseed oil or supplementation of nitrate. Linseed oil (Battle Hayward & Bower, Lincoln, UK) was included at 4% and nitrate in the form of Calcinit™ (Yara, Oslo, Norway; containing 75% nitrate in DM) at 2% (both on a DM basis). Diets were formulated to be *iso*-nitrogenous and similar in non-protein nitrogen and calcium through the addition of urea and limestone, respectively, to the control and linseed oil diet (Table 1). In Dairy 2 diets not containing linseed oil were supplemented with a rumen inert fat source (ButterfatExtra™, Trident, UK) to additionally balance the diets for fat content. Animals in both experiments were slowly adapted to the nitrate in the feed to prevent nitrite accumulation in the rumen and the potential risk of the occurrence of methemoglobinemia [7]. Animals receiving the nitrate diet were fed 25% of the final dose for the first two days, and thereafter the dose was increased by 25% of the final dose every two days. On day 7 and thereafter of each period the final dose of 2% nitrate was offered. Linseed oil was introduced at 4% from day 1 of each period.

### Blood sampling

Blood was taken from the tail vessels 3 h after feeding in both experiments. Samples were collected in heparinized evacuated tubes (Vacutainers, Becton Dickinson, Franklin Lakes, NJ, US) and immediately placed on ice. In Dairy 1, blood samples were taken at day 5 of the measurement period and analyzed within 1 h. In Dairy 2 samples were taken weekly, from day 3 of the first period onwards for all cows until the end of the experiment and analyzed within 30 min. Blood samples were analyzed for hemoglobin and methemoglobin (MetHb) using a blood gas analyzer (ABL800 in Dairy 1 and ABL700 in Dairy 2, Radiometer, Copenhagen, Denmark).

### Milk sampling

In Dairy 1, all 4 milkings when animals were in chambers were sampled. In Dairy 2, 4 milkings in total from day 29 and 31 (pm and am) of each period (during gaseous emissions measurement) were sampled and stored at 4°C. Milk constituents (protein, fat, lactose) and Somatic Cell Count (SCC) in both experiments were determined using mid infrared (MIR) spectroscopy at external laboratories.

### Feed sampling

During the measurement periods of both experiments, 600 g samples were taken from each prepared TMR and feed refusals were collected daily. Feed ingredients were sampled twice weekly during TMR preparation. Subsamples were taken for DM analysis and the remainder was stored at -20°C until further analysis. In Dairy 1, TMR samples from each treatment were pooled per chamber period (4 samples) at the end of the experiment. In Dairy 2, all TMR samples were pooled by treatment and period. Pooled samples were freeze dried and ground in a hammer mill to pass a 1mm^2^ screen before analysis. Samples were analyzed for gross energy (GE) by bomb calorimetry, for nitrogen by total combustion (AOAC 968.06), crude ash by combustion at 550°C (AOAC 942.05) and NDF (AOAC 2002.04) and ADF (AOAC 973.18). Crude fat in Dairy 1 was analyzed by Soxtec extraction (AOAC 991.36) and for Dairy 2 by hydrolysis and Soxhlet extraction (ISO 6492; [8]). Starch in Dairy 1 was analyzed by α-amylase method (AOAC 996.11) and for Dairy 2 by polarimetry according to Ewers (ISO 6493; [9]). Nitrate in feed in Dairy1 was analyzed according to a modified flow injection method (AOAC 968.07) and for Dairy 2 by ion chromatography as described in van Zijderveld et al. [10].

### Gaseous emissions measurements

In Dairy 1 gaseous emissions were measured starting on day eight of the measurement period before the morning feeding for two consecutive 24h periods. Emissions were measured from each animal individually in open circuit respiration chambers. Animals were adapted to the environment of the chamber by restraining them in the individual pens for short periods two days prior to the chamber measurements. The chambers were 4 m long × 2m wide × 2.2 m high and a larger version of the sheep respiration chambers in the same facility described in detail by Pinares-Patiño et al. [11]. Air flow through each chamber was 1.8 m^3^/min. Animals in Dairy 2 were moved to the chambers on day 28 of each period before the morning feeding. Gaseous emissions were measured from each animal individually for five consecutive 24 h periods in open circuit respiration chambers). Animals were restrained within the chambers in yokes, similar to the housing during the rest of the experimental periods. The chambers were 3.3 m long × 2.4 m wide × 2.4 m high and a larger version of the small ruminant respiration chambers at Aberystwyth University described in detail by Hart et al. [12]. Air flow through each chamber was 6.0 m^3^/min. In both experiments respiration chambers were opened (for approximately 45 min) twice daily for milking, cleaning, feed refusal collection and feeding. Gas measurements recorded during the time the chambers were open were excluded.

Methane emissions are expressed as methane emissions (g/d), methane yield (g/kg DMI) and methane intensity (g/kg fat and protein corrected milk (FPCM calculate as Milk Yield * (0.0337 + 0.116 * fat (%) / 10.3 + 0.06 * protein (%) / 10.3) *10)

### Rumen sampling

For Dairy 1, rumen samples were taken via stomach tube 3 h after morning feeding on day 5 and 6 during the measurement period. A sample of approximately 40 mL was taken from which aliquots were taken for VFA and ammonia analysis. The remainder of the rumen sample was stored at -20°C for microbial population analysis. For Dairy 2, rumen samples were taken on day 25 and 26 of each period. Samples were taken just before feeding (0 h) and at 0.5, 1, 2, 4, 6 and 8 h after feeding. Samples were collected manually through the rumen cannula using a 50 cm long tube with multiple 3 mm holes at the end of the tube located at the center of the rumen. At all the time points, a 50 mL sample was drawn from the end of the tube with a syringe. Rumen fluid pH was recorded immediately after sample collection and aliquots were taken for VFA, ammonia, and nitrate and nitrite analysis. At 2 h after feeding samples from the solid and liquid fractions of the rumen contents were taken for microbial population analysis. The samples were mixed by hand and then strained through 2 layers of muslin to separate the liquid and solid fraction. Aliquots of both the solid and liquid fractions were immediately frozen separately in liquid nitrogen. Samples were transported on dry ice to the lab and stored at -80°C. Rumen fluid samples from Dairy 1 taken for VFA and ammonia analysis were analyzed according to Wang et al. [13]. Rumen fluid samples from Dairy 2 were analyzed for VFA, ammonia according to de la Fuente et al. [14] and nitrate and nitrite according Miranda et al. [15].

### DNA extraction and quantitative PCR

Rumen samples from both experiments were freeze dried and DNA was extracted using the Qiagen mini stool kit (Qiagen Ltd., West Sussex, UK) as described in Skřivanová et al. [16]. The abundance of bacteria, archaea, fungi and protozoa was measured by determining the number of targeted sequences by qPCR using targeted primers (S1 Table) using reaction conditions and standards as described in Belanche et al. [17] expressed as known numbers of gene copies according to Dhanasekaran et al.[18].

### Ion-Torrent Next Generation Sequencing

Template DNA (75 ng/μL) was used to amplify the V1-V2 region of the bacterial or archaeal 16S rRNA gene. Samples were amplified with primers described in supplemental Table 1. Conditions for amplification of the bacterial and archaeal samples were similar to de la Fuente et al. [14] with the following changes to the reaction mix buffer and enzymes. For bacterial samples the reaction solution contained a reaction buffer (AccuBuffer 10X, Bioline, London, UK) and 1 μL of 2.5 U/μl of FastStart high fidelity enzyme (ACCUZYME™ DNA Polymerase, Bioline, London, UK). For the archaeal samples KAPA HiFi HotStart ReadyMix (2X; KAPAbiosystems, Boston, MA, United States) was used as the reaction mix and the number of cycles was increased to 30 cycles. Amplicon fragments were verified and purified as described before [14].

The emulsion PCR was carried out using the Ion PGM Template OT2 400 Kit (Life Technologies, Carlsbad, CA, United States) as described in the user guide (Catalog number: 4479878, Revision 2.0) provided by the manufacturer. Sequencing of the amplicon libraries was carried out on the Ion Torrent Personal Genome Machine (PGM) system using the Ion PGM Sequencing 400 Kit v2 (Life Technologies) following the corresponding protocol (Catalog number: 4482002, Revision 2.0). Raw sequence reads of all samples were deposited at the EBI Short Read Archive (SRA) from the European Nucleotide Archive (ENA) and can be accessed under the study accession numbers PRJEB10579 and PRJEB10640. Sequences were trimmed at 300 bp, filtered on quality (expected error < 1.5) and chimeras were removed before clustering into OTUs at 97% identity using USEARCH [19]. Samples were normalized to the lowest number of sequence in any sample based on random subsampling using Daisychopper (http://www.festinalente.me/bioinf/) and averaged across the two consecutive sampling days with OTUs present in only one day being discarded. OTUs were classified using RDP [20] and RIM-DB [21] for bacteria and archaea, respectively with a confidence limit for taxonomic classification of 50%.

### Statistical analysis

Parameters in both experiments were analyzed by the mixed model procedure in SAS 9.3 (SAS Institute Inc., Cary, NC, USA). For Dairy 1, data were analyzed according to the following model:

$$Y_{ijk}=\;\mu \;+\;T_{i}\;+B_{j}\;+\;e_{ijk}$$

Treatment (T) was a fixed effect and Block (B) was a random effect. Data from the replicated Latin square experiment of Dairy 2 were analyzed according to the following model:

$$Y_{ijkl}=\;\mu \;+\;S_{i}\;+C{\left(S\right)}_{ij}+\;P{\left(S\right)}_{kj}+\;T_{l}\;+e_{ijkl}$$

Square (S), period (P) within square and cow (C) within square were set as random variables, whereas treatment was a fixed variable. Period was nested in square since the periods of the two sets of 3 cows were started a week apart. Hourly rumen measurements were analyzed by including a repeated measures ANOVA in the mixed model procedure where hour was set as a repeated measure. Significance was declared at P≤0.05 and P≤0.10 was declared as a trend. Where treatment effect was significant, a pairwise comparison was performed with Tukey adjustment to describe differences between treatments. Microbial population analysis including rarefaction curves, diversity indexes and clustering methods were performed using the *R* statistical package (version 2.15; http://www.r-project.org/) using the ‘vegan’ package.

## Results

### Diet, animal performance and gaseous emissions

In Dairy 1, crude fat concentration was higher in the linseed oil diet (Table 1) and whilst there was an attempt to balance this in Dairy 2 the control diet still had a lower crude fat concentration than the linseed oil or nitrate diets. Analyzed nitrate concentration in the nitrate diet in Dairy 1 was 14 g/kg DM, which was lower than formulated, whereas the nitrate diet in Dairy 2 contained 20.3 g/kg DM, close to that formulated (20 g/kg DM). All diets were similar in CP and calcium and sulphur concentration were above requirements in all diets [22]. Dry matter intake was not different between treatments in Dairy 1 where cows were restricted in feed intake, but showed a tendency to be reduced in nitrate supplemented cows compared with the control cows in Dairy 2 (P = 0.054; Table 2). Milk yield, but not FCPM, was significantly increased for the nitrate treatment compared with the control in Dairy 1, but no significant differences were observed in Dairy 2 (Table 2). Milk fat percentage was decreased in linseed supplemented cows in both experiments (P<0.005), protein percentage also decreased in Dairy 2 (P = 0.009) with a tendency (P = 0.08) in Dairy 1. FPCM was reduced (P = 0.007) with linseed treatment compared with nitrate in Dairy 1, but not in Dairy 2 (Table 2). Hemoglobin and MetHb concentrations at 3 h post feeding were not different between treatments in Dairy 1 or Dairy 2 (Table 2). Maximum recorded values of MetHb with nitrate treatment were 13.8% and 6.2% for Dairy 1 and Dairy 2, respectively (Table 2), which are values considered not to cause clinical problems [7].

**Table 2:** Effect of dietary linseed oil and nitrate supplementation on DMI, milk yield, milk constituents, gaseous emissions and blood parameters of lactating dairy cows in Dairy 1 and Dairy 2.

	Dairy 1					Dairy 2				
	Control	Linseed oil	Nitrate	SEM	P	Control	Linseed oil	Nitrate	SEM	P
DMI, kg/d	18.4	17.6	18.3	0.91	0.26	16.6^A^	16.4^AB^	15.2^B^	0.51	0.06
Milk yield, kg/d	16.4^b^	17.0^ab^	19.5^a^	1.25	0.046	18.4	18.3	17.0	1.82	0.46
Fat, %	5.46^a^	3.38^b^	5.23^a^	0.277	<0.001	5.77^a^	4.47^b^	6.14^a^	0.414	0.004
Protein, %	4.28	3.92	3.89	0.162	0.08	3.40^a^	3.17^b^	3.41^a^	0.132	0.009
Lactose, %	5.19	5.2	5.23	0.081	0.94	4.63^ab^	4.77^a^	4.52^b^	0.150	0.023
SSC, x1000/mL	90	146	65	42.2	0.40	88	51	119	35.3	0.25
FPCM, kg/d	18.7^ab^	15.3^b^	21.1^a^	1.12	0.007	20.7	18.0	20.6	1.94	0.42
**Emissions**										
Methane, g/d	394^a^	376^ab^	317^b^	21.6	0.020	430^a^	417^a^	337^b^	15.0	0.001
Methane yield, g/kg DMI	21.3^a^	21.5^a^	17.4^b^	0.88	0.011	25.9^a^	25.5^a^	22.4^b^	0.72	0.006
Methane intensity, g/kg FPCM	21.2^a^	25.0^a^	15.2^b^	1.62	<0.001	21.4^ab^	23.7^a^	17.5^b^	2.22	0.039
Hydrogen, g/d	0.7^b^	1.1^ab^	2.9^a^	0.54	0.027	1.0^b^	1.1^b^	3.5^a^	0.20	0.001
**Blood parameters**										
Hemoglobin, g/L	108	105	105	4.7	0.69	91	87	90	0.24	0.16
Methemoglobin, % Hb	1.8	2.2	3.6	0.80	0.10	1.2	1.2	2.1	0.41	0.24

DMI, dry matter intake; FPCM, fat and protein corrected milk; Hb, hemoglobin; SSC, somatic cell count

^a,b^ LS means with different letter in superscripts are different at P < 0.05.

^A,B^ LS means with different letter in superscripts are different at P < 0.10.

Nitrate supplementation decreased methane emissions per day (P<0.02), methane yield (g/kg DMI; P<0.01) and methane intensity (in g/kg FPCM; P<0.04) in both experiments (Table 2). Daily hydrogen emissions increased with nitrate supplementation (P<0.03) compared with the control diet in both experiments. The linseed oil treatment did not affect emissions of either methane or hydrogen in either experiment (Table 2).

### Rumen fermentation

Total rumen VFA concentration was not different between treatments in either experiment (Table 3). The proportion of propionate was decreased with the nitrate treatment compared with the control in both experiments (P<0.02) and hence the acetate to propionate ratio increased in nitrate supplemented cows compared with the controls in Dairy 1 (P = 0.023) and in Dairy 2 (P<0.001). The proportion of *n*-butyrate increased (P = 0.041) with linseed oil supplementation compared with the control in Dairy 1 and with nitrate supplementation (P = 0.002) compared with the other treatments in Dairy 2. Rumen pH was only measured in Dairy 2 and was not different between treatments (Table 3). Nitrate and nitrite concentrations in rumen fluid were significantly (P<0.05) increased in the nitrate treatment in Dairy 2 compared with the control and linseed treatment (Table 3).

**Table 3:** Effect of dietary linseed oil and nitrate supplementation on rumen fermentation of lactating dairy cows in Dairy 1 and Dairy 2.

	Dairy 1					Dairy 2						
	Control	Linseed oil	Nitrate	SEM	P trt	Control	Linseed oil	Nitrate	SEM	P trt	P time	P trt x time
pH	ND	ND	ND	-	-	6.03	6.01	6.20	0.093	0.33	<0.001	0.026
Ammonia, mM	23.3	21.7	19.1	2.43	0.49	4.61	5.16	4.47	0.284	0.22	<0.001	0.91
Nitrate, μM	ND	ND	ND	-	-	7^b^	8^b^	123^a^	28.2	0.015	0.006	<0.001
Nitrite, μM	ND	ND	ND	-	-	3^b^	4^b^	110^a^	29.3	0.042	0.003	<0.001
Total VFA, mM	100.3	91.0	94.5	4.88	0.35	128.6	138.4	127.7	6.63	0.26	0.011	0.59
**Individual VFAs, mol/100 mol**												
Acetate	62.8^B^	62.4^B^	66.3^A^	1.20	0.07	54.7	53.0	56.7	1.15	0.11	<0.001	0.06
Propionate	20.4^a^	17.7^ab^	15.3^b^	1.02	0.016	20.9^a^	21.6^a^	16.8^b^	0.75	<0.001	<0.001	0.50
*n*-Butyrate	11.6^b^	14.5^a^	13.2^ab^	0.74	0.041	16.4^b^	17.2^b^	18.4^a^	0.33	0.002	0.18	<0.001
*n*-Valerate	1.5	1.6	1.4	0.05	0.22	2.6	2.6	2.4	0.10	0.33	<0.001	0.12
Acetate: Propionate ratio	3.16^b^	3.55^ab^	4.51^a^	0.27	0.013	2.66^b^	2.48^b^	3.40^a^	0.142	<0.001	<0.001	0.62

Rumen in Dairy 1 was sampled at 3 h after morning feeding and in Dairy 2 at 0.5, 1, 2, 4, 6 and 8 h after feeding. ND, not determined; trt, treatment, VFA, volatile fatty acid.

^a,b^ LS means with different letter in superscripts are different at P < 0.05.

^A,B^ LS means with different letter in superscripts are different at P < 0.10.

### Microbial community analysis

Rumen Bacterial 16S gene copy number (log_10_ copies per g DM) showed a tendency (P = 0.054; Table 4) to be decreased with linseed oil supplementation compared with the control in the solid phase in Dairy 2, whereas archaeal 16S gene copy number was significantly decreased (P = 0.019) with nitrate supplementation in the solid phase in Dairy 2. Fungal 18S/ITS copy number was decreased (P = 0.008) in the linseed treatment for the mixed rumen sample in Dairy 1. Overall, number of copies of the target genes for bacteria, archaea and fungi, but not protozoa, were higher in the solid phase of rumen contents, compared with the liquid phase in Dairy 2.

**Table 4:** Effect of dietary linseed oil and nitrate supplementation on number of bacteria, archaea, protozoa and fungi in the rumen of lactating dairy cows in Dairy 1 and Dairy 2.

	Dairy 1						Dairy 2					
	Phase	Control	Linseed oil	Nitrate	SEM	P	Phase	Control	Linseed oil	Nitrate	SEM	P
Bacteria, log_10_ 16S	Mix	11.43	11.14	11.26	0.156	0.34	Liquid	11.56	11.35	11.19	0.117	0.13
copies/g DM^1^							Solid	11.84^A^	11.76^B^	11.80^AB^	0.021	0.06
Archaea, log _10_ mcrA	Mix	8.42	8.22	8.10	0.291	0.64	Liquid	8.99	8.73	8.48	0.176	0.15
copies/g DM							Solid	9.33^a^	9.21^ab^	9.11^b^	0.065	0.019
Protozoa, log_10_ of 18S	Mix	9.62	10.33	10.04	0.298	0.14	Liquid	10.27	10.17	10.32	0.133	0.74
copies/g DM							Solid	10.15	10.22	10.15	0.092	0.81
Fungi, log_10_ of 18S/ITS	Mix	6.62^a^	5.43^b^	6.45^a^	0.376	0.008	Liquid	7.41	7.22	7.20	0.148	0.57
copies/g DM							Solid	8.35	8.34	8.41	0.082	0.79

Number of gene copies are log transformations and expressed per g of rumen. DM content; DM, dry matter.

^1^ Data were transformed (log) to obtain homogeneity of variance.

^a,b^ LS means with different letter in superscripts are different at P < 0.05.

^A,B^ LS means with different letter in superscripts are different at P < 0.10.

For the bacteria samples, in total 10,578,125 sequences containing a barcode were submitted to the UPARSE pipeline, from which 4,175,098 high quality sequences were obtained belonging to 4,190 OTUs. Data were normalized to 6,767 sequences per sample. Rarefaction curves (S1 Fig) for the bacteria samples showed that a plateau was not reached, indicating that complete sampling of these environments had not been achieved.

For the archaeal samples, in total 1,890,139 sequences containing a barcode were submitted to the UPARSE pipeline, from which 580,406 high quality sequences were obtained belonging 143 OTUs. Data were normalized to 4,078 sequences per sample. For the archaea libraries a total of 112 OTUs were assigned to bacterial taxa OTUs (using RDP) and these OTUs were discarded (4.1% of total sequences) prior to using RIM-DB to classify the remaining archaeal sequences.

Total number of bacterial OTUs was not different between treatments (Table 5). The higher number of OTU in the solid phase compared with the liquid phase also resulted in numerically higher Shannon’s and Simpson’s diversity indexes. Archaeal diversity was much lower than bacterial diversity. A tendency (P = 0.09) for a higher number of OTUs in the control treatment compared with the other treatments was observed in the liquid phase of Dairy 2. A tendency (P = 0.08) for a higher diversity in the linseed oil treatment compared with the nitrate treatment was observed in Dairy 1 using the Simpson’s diversity index.

**Table 5:** Effect of dietary linseed oil and nitrate supplementation on diversity indexes of the rumen bacterial and archaeal populations in the rumen of lactating dairy cows in Dairy 1 and Dairy 2.

	Dairy 1						Dairy 2					
	Phase	Control	Linseed oil	Nitrate	SEM	P	Phase	Control	Linseed oil	Nitrate	SEM	P
**Bacteria**												
Number of OTU	Mix	795	737	754	25.2	0.25	Liquid	707	739	715	40.7	0.85
							Solid	915	914	861	39.0	0.55
Shannon’s, *H*	Mix	5.76	5.77	5.66	0.099	0.57	Liquid	5.56	5.75	5.68	0.134	0.61
							Solid	6.07	6.13	5.98	0.108	0.61
Simpson’s, 1-*D*	Mix	0.987	0.993	0.988	0.0029	0.23	Liquid	0.987	0.993	0.992	0.0027	0.34
							Solid	0.994	0.996	0.993	0.0016	0.58
**Archaea**												
Number of OTU	Mix	25.5	25.2	25.5	0.39	0.76	Liquid	24.2^A^	22.6^B^	22.8^AB^	0.89	0.09
							Solid	22.0	20.2	21.0	0.75	0.27
Shannon’s, *H*	Mix	2.50	2.62	2.45	0.064	0.19	Liquid	1.87	1.87	1.96	0.138	0.83
							Solid	2.00	1.92	1.88	0.079	0.44
Simpson’s, 1-*D*	Mix	0.886^AB^	0.909^A^	0.874^B^	0.0105	0.08	Liquid	0.732	0.748	0.755	0.0432	0.79
							Solid	0.799	0.772	0.769	0.0225	0.53

Mixed rumen content in Dairy 1 was obtained by stomach tube at 3 h after morning feeding and cows in Dairy 2 were samples through a rumen cannula at 2 h after feeding and samples were split by liquid and solid phase. OTU, operational taxonomic unit.

^A,B^ LS means with different letter in superscripts are different at P < 0.10.

**Fig 1:**
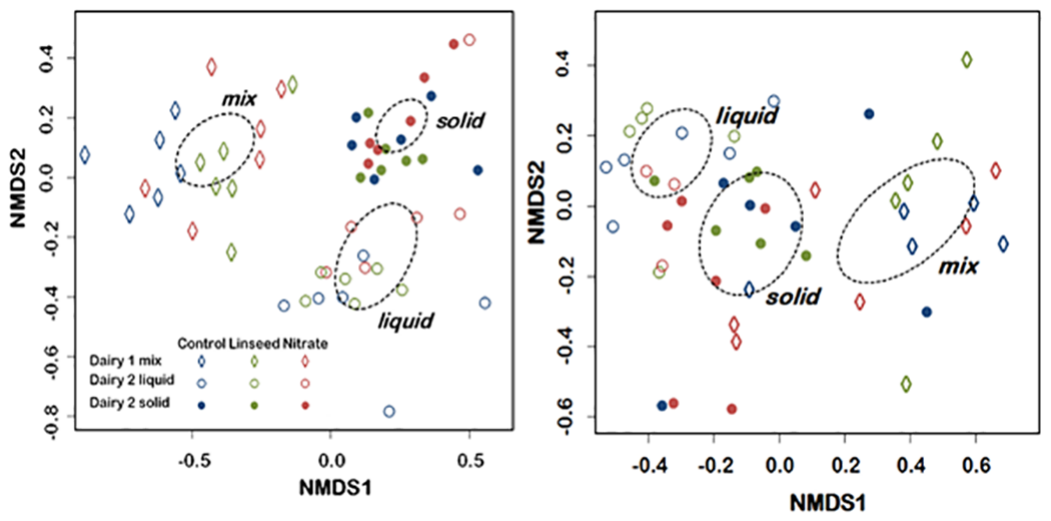
Non-metric dimensional scaling (NMDS) plot of the first two scaling components from rumen bacterial (A) and archaeal (B) communities analysed with NGS techniques of lactating dairy cows supplemented with dietary linseed oil or nitrate. Mixed rumen content in Dairy 1 was obtained by stomach tube at 3 h after morning feeding and cows in Dairy 2 were samples through a rumen cannula at 2 h after feeding and samples were split by liquid and solid phase. Ellipses indicate the 99% confidence interval based on SE around the phase centroids.

Non-metric dimensional scaling (NMDS) analysis of the bacterial community revealed clustering towards liquid versus solid phase and experiment, with samples from Dairy 1 and Dairy 2 clustering completely apart (Fig 1A). Clustering towards treatment was not apparent. For the archaeal community, NMDS analysis also showed clustering towards phase and experiment, but this was less apparent than for the bacterial samples as samples from Dairy 1 and Dairy 2 did overlap (Fig 1B). Clustering towards treatment was not apparent in Dairy 2 samples, but some clustering by treatment was apparent in Dairy1 samples, although samples did overlap (Fig 1B).

Relative abundances of bacterial genera present at 0.2% abundance or above are presented in Table 6. On average, almost 60% of OTUs could not be identified at genera level. *Prevotella* (*Prevotellaceae* family) was the most abundant genus (293 OTUs assigned accounting for 23.6% of sequences in both experiments). Nitrate treatment significantly decreased the relative abundance of *Prevotella* in all three datasets (Table 6). *Succiniclasticum* (*Acidaminococcaceae* family) was the most abundant genus in the *Firmicutes* phylum and was significantly increased in the linseed oil treatment compared with the control in Dairy 1. *Phocaeicola* (family of uncertain placement in the *Bacteroidetes* phylum) was significantly affected by treatment in all three datasets (P<0.02), where nitrate treatment increased relative abundance compared with linseed oil. *Fibrobacter* (*Fibrobacteraceae* family) was significantly increased in the nitrate treatment in the solid phase of Dairy 2. *Barnesiella* (*Porphyromonadaceae* family) was increased (P = 0.006) in the linseed oil treatment compared with the other treatments in Dairy 1, whereas nitrate treatment decreased its abundance in the solid phase of Dairy 2. The genus of uncertain placement within the SR1 phylum (SR1 *genus insertae sedis*) was decreased (P = 0.006) in the linseed oil treatment compared with the nitrate treatment for all three datasets and also compared with the control treatment in Dairy 1. *Anaerovorax* (*Clostridiales incertae sedis XIII* family) was increased (P = 0.035) in the nitrate treatment compared with linseed oil supplementation in the liquid phase of Dairy 2, but decreased with nitrate supplementation in the solid phase of Dairy 2 (P = 0.003). Furthermore significant treatment differences were observed for *Mogibacterium*, *Paraprevotella* (*Prevotellaceae* family) *Syntrophococcus* (*Lachnospiraceae* family), *Solobacterium* (*Erysipelotrichaceae* family), but these were only observed in one of the datasets and represent a small part of the population.

**Table 6:** Effect of dietary linseed oil and nitrate supplementation on relative abundance of bacteria at genera level above 0.2% average abundance in the rumen of lactating dairy cows in Dairy 1 and Dairy 2.

	Dairy 1						Dairy 2					
	Phase	Control	Linseed oil	Nitrate	SEM	P	Phase	Control	Linseed oil	Nitrate	SEM	P
*Prevotella*, %	Mix	20.3^ab^	26.3^a^	17.9^b^	2.12	0.010	Liquid	37.0^a^	38.8^a^	26.1^b^	2.59	0.008
							Solid	16.4^a^	17.0^a^	12.4^b^	1.17	0.003
*Succiniclasticum*, %	Mix	1.2^b^	2.3^a^	1.4^ab^	0.22	0.017	Liquid	2.2	2.6	2.1	0.21	0.28
							Solid	1.6	1.7	1.5	0.16	0.72
*Phocaeicola*, %	Mix	3.0^a^	1.2^b^	2.4^a^	0.37	0.002	Liquid	1.2^ab^	1.0^b^	1.7^a^	0.15	0.018
							Solid	1.0^b^	1.0^b^	1.5^a^	0.10	0.009
*Fibrobacter*, %	Mix	0.57^AB^	0.43^B^	1.29^A^	0.252	0.08	Liquid	0.86^B^	1.23^AB^	1.54^A^	0.169	0.08
							Solid	1.46^b^	2.66^ab^	3.05^a^	0.325	0.022
*Hallela*, %	Mix	0.23	0.59	0.58	0.129	0.13	Liquid	0.84	1.14	2.39	0.465	0.12
							Solid	2.08	2.16	2.47	0.304	0.65
*Ruminococcus*, %	Mix	1.14	0.95	0.64	0.179	0.17	Liquid	0.54	0.54	0.68	0.122	0.67
							Solid	2.38^A^	2.35^AB^	1.97^B^	0.154	0.06
*Mogibacterium*, %	Mix	0.72^b^	1.78^a^	1.31^ab^	0.320	0.008	Liquid	0.21	0.21	0.39	0.057	0.11
							Solid	1.71	1.80	1.83	0.190	0.88
*Ruminobacter*, %	Mix	2.54	1.14	2.16	1.36	0.64	Liquid	0.75	0.41	0.51	0.154	0.34
							Solid	0.52	0.18	0.19	0.144	0.24
*Paraprevotella*, %	Mix	0.37	0.46	0.33	0.072	0.45	Liquid	0.99	1.14	0.86	0.207	0.65
							Solid	0.45^ab^	0.54^a^	0.35^b^	0.056	0.012
*Anaeroplasma*, %	Mix	0.95	0.85	0.75	0.118	0.44	Liquid	0.62	0.31	0.76	0.133	0.12
							Solid	0.47	0.28	0.38	0.101	0.47
*Barnesiella*, %	Mix	0.57^b^	1.4^a^	0.57^b^	0.174	0.006	Liquid	0.51^A^	0.39^B^	0.43^AB^	0.056	0.06
							Solid	0.50^a^	0.34^ab^	0.31^b^	0.063	0.042
*SR1 (genera incertae*	Mix	1.50^a^	0.20^b^	1.43^a^	0.247	0.006	Liquid	0.17^ab^	0.04^b^	0.33^a^	0.077	0.005
*sedis)*, %							Solid	0.22^ab^	0.02^b^	0.40^a^	0.085	0.016
*Aquiflexum*, %	Mix	0.49	0.37	0.38	0.120	0.76	Liquid	0.27	0.49	0.34	0.117	0.42
							Solid	0.28	0.54	0.39	0.103	0.27
*Anaerovorax*, %	Mix	0.26	0.29	0.29	0.054	0.93	Liquid	0.16^ab^	0.11^b^	0.20^a^	0.023	0.035
							Solid	0.71^a^	0.78^a^	0.63^b^	0.043	0.003
*Butyrivibrio*, %	Mix	0.23^B^	0.36^A^	0.25^AB^	0.070	0.10	Liquid	0.21	0.24	0.28	0.066	0.77
							Solid	0.40	0.55	0.57	0.061	0.20
*Lachnospiracea*	Mix	0.14	0.12	0.11	0.025	0.78	Liquid	0.21	0.19	0.22	0.032	0.75
*(incertae sedis)*, %							Solid	0.66	0.77	0.55	0.077	0.20
*Syntrophococcus*, %	Mix	0.15^ab^	0.25^a^	0.13^b^	0.039	0.020	Liquid	0.10	0.12	0.17	0.038	0.42
							Solid	0.57	0.75	0.67	0.054	0.15
*Succinivibrio*, %	Mix	0.04^B^	0.02^B^	0.65^A^	0.021	0.09	Liquid	0.48	0.46	0.33	0.132	0.70
							Solid	0.20	0.24	0.10	0.076	0.41
*Succinimonas*, %	Mix	0.19	0.32	0.49	0.245	0.69	Liquid	0.31	0.34	0.32	0.126	0.98
							Solid	0.17	0.13	0.18	0.082	0.91
*Solobacterium*, %	Mix	0.27	0.14	0.25	0.046	0.17	Liquid	0.07^ab^	0.06^b^	0.17^a^	0.027	0.042
							Solid	0.41	0.44	0.52	0.050	0.33
*Sporobacter*, %	Mix	0.51	0.42	0.42	0.113	0.77	Liquid	0.14	0.10	0.15	0.030	0.34
							Solid	0.22	0.17	0.16	0.029	0.25
*Sediminitiomix*, %	Mix	0.47	0.39	0.11	0.149	0.16	Liquid	0.30	0.40	0.04	0.014	0.22
							Solid	0.09	0.13	0.00	0.048	0.24
*Sharpea*, %	Mix	0.47	0.33	0.36	0.216	0.89	Liquid	0.09	0.00	0.13	0.068	0.41
							Solid	0.25	0.02	0.20	0.127	0.44
*Clostridium IV*, %	Mix	0.10	0.28	0.09	0.072	0.17	Liquid	0.18	0.09	0.14	0.048	0.41
							Solid	0.22	0.14	0.14	0.049	0.45

Mixed rumen content in Dairy 1 was obtained by stomach tube at 3 h after morning feeding and cows in Dairy 2 were samples through a rumen cannula at 2 h after feeding and samples were split by liquid and solid phase.

^a,b^ LS means with different letter in superscripts are different at P < 0.05.

^A,B^ LS means with different letter in superscripts are different at P < 0.10.

All archaeal OTUs classified as the phylum of *Euryarchaeota* and could be classified to genus level (Table 7). *Methanobrevibacter* (*Methanobacteriaceae* family) was the most abundant genus, followed by Group 12 of the *Methanomassiliicoccaceae* family and *Methanosphaera (Methanobacteriaceae* family). Within the *Methanomassiliicoccaceae* family an increase (P = 0.003) in Group 3a was observed with nitrate supplementation in Dairy 1. A decrease in Group 8 within the same family was observed in the nitrate treatment in the solid phase in Dairy 2 and also in the linseed treatment in the solid phase compared with the control.

**Table 7:** Effect of dietary linseed oil and nitrate supplementation on relative abundance of archaea at genus level above 0.2% average abundance in the rumen of lactating dairy cows in Dairy 1 and Dairy 2.

	Dairy 1						Dairy 2					
	Phase	Control	Linseed oil	Nitrate	SEM	P	Phase	Control	Linseed oil	Nitrate	SEM	P
*Methanobacteriaceae*												
*Methanobrevibacter*, %	Mix	48.7	56.7	40.3	5.38	0.12	Liquid	33.0	39.3	35.8	7.70	0.63
							Solid	51.6	40.4	43.0	3.92	0.16
*Methanosphaera*, %	Mix	8.4^AB^	12.9^A^	7.7^B^	1.54	0.06	Liquid	2.3	3.0	4.1	0.58	0.11
							Solid	7.9	7.3	7.6	0.88	0.88
*Methanomassiliicoccaceae*												
*Group 12*, %	Mix	24.8	19.0	31.8	5.24	0.23	Liquid	60.5	56.8	55.9	6.09	0.74
							Solid	38.5	51.5	47.4	4.42	0.13
*Group 3a*, %	Mix	9.5^b^	6.6^b^	13.9^a^	1.21	0.003	Liquid	0.94	0.00	1.50	0.606	0.19
							Solid	0.45	0.21	0.94	0.267	0.18
*Group 3b*, %	Mix	6.2^A^	3.9^B^	4.5^AB^	0.73	0.08	Liquid	0.30	0.09	0.36	0.118	0.20
							Solid	0.04	0.02	0.09	0.035	0.33
*Group 10*, %	Mix	0.9	0.2	0.5	0.52	0.70	Liquid	1.2	0.0	1.4	1.49	0.60
							Solid	0.8	0.2	0.5	0.56	0.45
*Group 11*, %	Mix	0.5	0.3	0.4	0.08	0.27	Liquid	0.7	0.7	0.6	0.19	0.78
							Solid	0.4	0.4	0.4	0.07	0.92
*Group 8*, %	Mix	0.7	0.4	0.7	0.14	0.25	Liquid	0.47^a^	0.27^ab^	0.11^b^	0.101	0.044
							Solid	0.08^a^	0.03^b^	0.01^b^	0.013	0.015
*Methanosarcinaceae*												
*Methanimicrococcus*, %	Mix	0.20^A^	0.01^B^	0.11^AB^	0.050	0.06	Liquid	0.44	0.02	0.25	0.232	0.23
							Solid	0.17	0.00	0.04	0.055	0.11

Mixed rumen content in Dairy 1 was obtained by stomach tube at 3 h after morning feeding and cows in Dairy 2 were samples through a rumen cannula at 2 h after feeding and samples were split by liquid and solid phase.

^a,b^ LS means with different letter in superscripts are different at P < 0.05.

^A,B^ LS means with different letter in superscripts are different at P < 0.10.

## Discussion

Methane yield was higher in Dairy 2 than Dairy 1, but both were in the range typically observed in dairy cows [23, 24]. Linseed oil did not affect methane emissions in either experiment. This contrasts with studies in the literature showing a decrease in methane emissions by around 3.6 to 5.6% for each 10 g/kg DM increase in crude fat content [23, 25–27]. Linseed oil is thought to decrease methane emissions as the PUFA present in linseed oil might be toxic to certain cellulolytic bacteria and rumen protozoa which supply hydrogen to the methanogenic archaea [27]. However, no effect was observed on the main cellulolytic genera such as *Fibrobacter* or *Ruminococcus* and neither did linseed oil decrease protozoa 18S copy numbers. Overall, the absence of a reduction in methane emissions with linseed oil coincides with an absence of a response in the rumen microbial ecosystem as a whole. The effects of linseed oil on animal performance, particularly on milk composition were, however, pronounced in both experiments. Linseed oil caused a reduction in milk fat percentage in agreement with previous studies (see meta-analysis of [28]). It is believed this is caused by the biohydrogenation of PUFA into specific FA intermediates, in particular *trans*-10, *cis*-12 conjugated linoleic acid which inhibit the *de novo* synthesis of FA in the mammary gland, causing a reduction in saturated FA and total milk fat [29].

In agreement with previous studies, nitrate caused a significant decrease in daily methane emissions, methane yield and methane intensity [10,30, 31] with the size of the decrease (20–22%) consistent with that reported previously [31, 32]. Based on formulated nitrate inclusion levels the complete reduction of nitrate to ammonia would decrease methane emissions by 5.2 g methane/kg of DMI. The observed decreases were 3.9 g or 76% of the potential in Dairy 1 and 3.5 g or 68% in Dairy 2, higher than observed in previous studies with lactating dairy cows, observing 59% of the theoretical potential [32]. Furthermore, in both experiments an increase in hydrogen emissions with nitrate feeding was observed consistent with previous studies [32]. However whilst the pattern of methane emissions over the day (S3 Fig) was similar to that reported previously [32] the pattern of hydrogen production was more variable in this study compared to previous observation [32] particularly in Dairy 1 with a notable peek in hydrogen after feeding (S4 Fig).

Although no clear effect of nitrate on animal performance was observed, nitrate decreased propionate proportion and hence acetate to propionate ratio in both experiments, which is in agreement with previous studies [30, 31, 33]. Nitrate reduction can compete with propionigenesis for reducing equivalents [34] as it is thermodynamically more favorable, diverting glucose fermentation away from the propionigenesis into acetate in Dairy1 and butyrate in Dairy 2. The decrease in the proportion of propionate could also partially explain the lower theoretical efficiency of nitrate as reducing equivalents are not diverted away from methanogenesis but from propionigenesis. Rumen nitrate and nitrite concentrations in Dairy 1 were not quantified but in Dairy 2 nitrate and nitrite concentrations only significantly increased at 0.5 and 1h post feeding (S2 Fig). This would suggest that nitrate was quickly reduced to nitrite and subsequently into ammonia. Consistent with this a there was no significant effect of nitrate on either hemoglobin nor MetHb which is contrast to the observations of an Zijderveld et al [32] who reported an increase in MetHb and decrease in hemoglobin in nitrate supplemented animals but agrees with the observations of Nolan et al. [31] and suggests that there is variation between trials in the conversation of nitrate to ammonia within the rumen.

In agreement with previous studies the total number of OTU in the bacterial community was much higher than in the archaeal community [35, 36]. *Methanobrevibacter* was the most abundant archaeal genus in all three datasets in line with other studies [35, 37]. In agreement with a previous *in vivo* study [10] archaeal numbers decreased with nitrate supplementation in the solid phase in Dairy 2. This may either be due to a lower availability of hydrogen due to nitrate and nitrite reducing microbes depleting the hydrogen pool or by direct toxicity of the intermediate nitrite [38, 39]. As nitrate decreased methane emissions, the acetate to propionate ratio increased in both studies and nitrate and nitrite concentrations decreased shortly after feeding in Dairy 2 (S2 Fig) it may well be that in our study part of the reduction in methanogen density can be attributed to the depletion of the hydrogen pool.

*Prevotella* was the most abundant bacterial genus in all three datasets, as previously reported in other rumen studies [40, 41]. In contrast to previous *in vitro* work [42] *Prevotella* abundance decreased in nitrate supplemented animals in all three datasets. Prevotella abundance has previously increased while decreasing methane emissions and increasing hydrogen emissions [3]. *Prevotella* spp. are metabolically versatile, capable of utilizing a wide variety of proteins, peptides and monosaccharides as well as plant polysaccharides [43, 44], although they are known to mainly degrade more readily fermentable carbohydrates [45]. Furthermore, *Prevotella spp*. are known to produce propionate as a major fermentation product [46] and increases in propionate proportions in the rumen have been associated with increases in *Prevotella in vivo* [3]. The decrease in propionate could therefore be linked to the decrease in *Prevotella* abundance. Whether nitrate or nitrite was toxic to members of the *Prevotella* genus or whether nitrate changed substrate availability, similar to the potential decrease in substrate (hydrogen) availability to the methanogenic archaea, is unknown.

*Fibrobacter* abundance tended to be increased or was significantly increased with nitrate supplementation. A well-known fiber degrading bacterium, *Fibrobacter succinogenes* does not produce hydrogen [45] unlike the other fiber degrading bacteria. Hence, the changes in fermentation and particular hydrogen dynamics with nitrate supplementation might have favored fermentation by *Fibrobacter*. Interestingly, of the genera known to include nitrate/nitrite reducing bacteria such as *Selenomonas ruminantium*, *Veillonella parvula* and *Wolinella succinogenes* [39, 47], only *Selenomonas* was observed, potentially harboring known nitrate and nitrate reducing species. Average abundance of the *Selenomonas* genus was below 0.02%, which lower than levels reported in some previous papers [48] but is consistent with levels found by qPCR in others [49], in agreement with previous studies [47] no differences were observed between treatments with only 20 out of 54 samples containing the genus. However, even in a nitrate-supplemented animal, the total abundance of known nitrate reducing species was found to be below 0.06% [47], either because they are rather efficient in the conversion of nitrate and nitrite, or because other rumen microbes also reduce nitrate and nitrate.

Linseed oil tended to increase archaeal species richness (Simpson’s index) in Dairy 1. These changes in population structure with linseed oil supplementation, however, did not affect methane emissions, whereas nitrate supplementation showed less of an effect on archaeal population structure, but did cause a reduction in total number of archaea and decreased methane emissions. Wallace et al [50] argued that archaeal number, rather than population structure is the major driver of methane production in the rumen, which is supported by the findings of this study.

The two experiments reported here were designed to investigate the variability in response to the dietary additives over geographical distances with measurement and sampling procedures were kept similar between experiments. To the authors’ knowledge this is the first study comparing the microbial community in the rumen of dairy cows in two different geographical regions, but receiving the same dietary supplements. However, some differences in procedure could have affected the results. First, in Dairy 1 linseed oil was included by substituting it for maize silage, creating a difference in crude fat concentration between diets, whereas in Dairy 2 diets were balanced for crude fat by adding a rumen inert fat source to the control and nitrate diets. Secondly, cows in Dairy 1 were restricted in feed intake during the measurement period, whereas cows in Dairy 2 were fed to allow for *ad libitum* intake. This created changes in the feed intake pattern, which is reflected in the diurnal gaseous emissions (S3 and S4 Figs). Thirdly, in terms of rumen sampling procedure for assessing the microbial population, DNA extraction onwards was identical between the two experiments, but the sampling technique was different. Collection of the rumen sample in Dairy 1 was by stomach tube, whereas in Dairy 2 samples were collected through the cannula. In a direct comparison, significant differences were observed in the relative abundance of bacteria, archaea, protozoa and fungi with sampling technique (cannula and stomach tube) and phase in dairy cows [40]. Nevertheless, here the same taxa were affected by dietary treatment, suggesting that even though the relative abundances in samples taken by stomach tube or liquid or solid phase samples taken through the fistula will differ, the shifts due to dietary treatment are similar and can be detected in all sample types. Furthermore, even though the samples from Dairy 1 and Dairy 2 were obtained from dairy cows of two geographically distinct regions and NMDS plots showed clustering by phase and experiment, the relative abundance at the genus level over the three datasets was relatively similar. No genera were found in only one dataset unless they were present in 4 or fewer samples, suggesting they were not part of the core microbiome in either experiment.

## Conclusions

This study has demonstrated that significant decreases in rumen methane emissions can be achieved without drastic effects on either the rumen microbial population or its function. Furthermore, we found no evidence to support concerns that the response to dietary additives in different geographical locations will differ due to different underlying rumen microbial populations.

## Supporting Information

S1 Fig Rarefaction curve showing sequencing depth of the bacterial (A) and archaeal community of rumen samples from lactating dairy cows supplemented with dietary linseed oil or nitrate. Lines represent normalized data of samples obtained through stomach tube (mixed rumen content or mix; green lines) in Dairy 1 or through a rumen cannula split by liquid (blue lines) or solid (red lines) fraction in Dairy 2.
(TIF)

S2 Fig Dairy 2; LS means of nitrate (A) and nitrite (B) concentrations of dairy cows supplemented with linseed oil or nitrate after morning feeding. Error bars indicate standard error.
(TIF)

S3 Fig Diurnal methane pattern of lactating dairy cows fed a control diet or diets containing linseed oil (4% of dietary DM) or nitrate (2% of dietary DM) in Dairy 1 (A) or Dairy 2 (B). Error bars indicate SE. Arrows indicate time of feeding.
(TIF)

S4 Fig Diurnal hydrogen pattern of lactating dairy cows fed a control diet or diets containing linseed oil (4% of dietary DM) or nitrate (2% of dietary DM) in Dairy 1 (A) or Dairy 2 (B). Error bars indicate SE. Arrows indicate time of feeding.
(TIF)

S1 Table Targeted primers used for quantification of bacteria, archaea, fungi and protozoa by qPCR and NGS. Fw, forward primer; NGS, next generation sequencing; qPCR, quantative PCR; Rev, reverse primer; T_a_, annealing temperature.
(DOCX)
