# Supplementary figures and images for: Does Dietary Mitigation of Enteric Methane Production Affect Rumen Function and Animal Productivity in Dairy Cows?
Source: PLoS One. 2015 Oct 28;10(10):e0140282. doi: 10.1371/journal.pone.0140282 (PMC4624802; doi:10.1371/journal.pone.0140282)

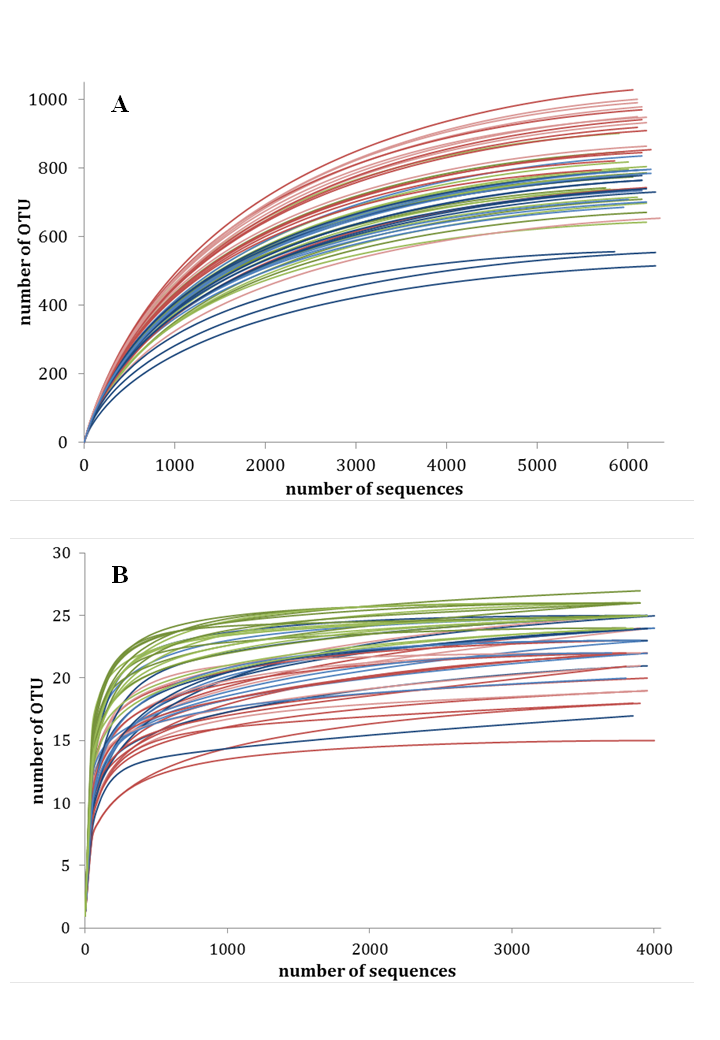

Supplement: S1 Fig — Lines represent normalized data of samples obtained through stomach tube (mixed rumen content or mix; green lines) in Dairy 1 or through a rumen cannula split by liquid (blue lines) or solid (red lines) fraction in Dairy 2. (TIF) [file pone.0140282.s001.tif]

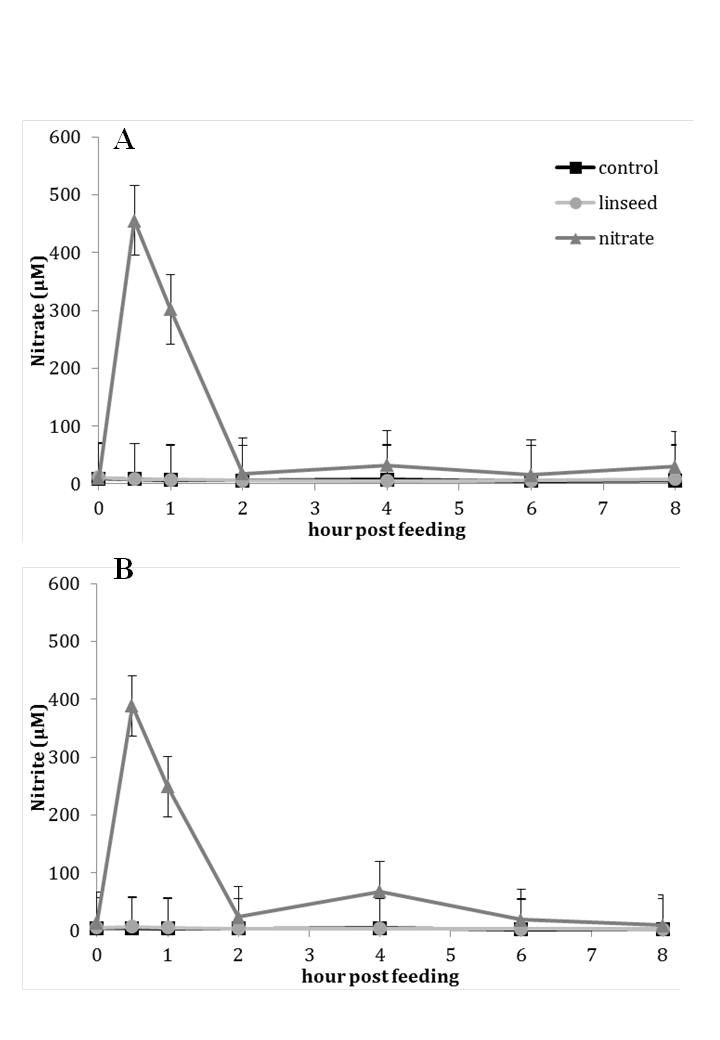

Supplement: S2 Fig — Error bars indicate standard error. (TIF) [file pone.0140282.s002.tif]

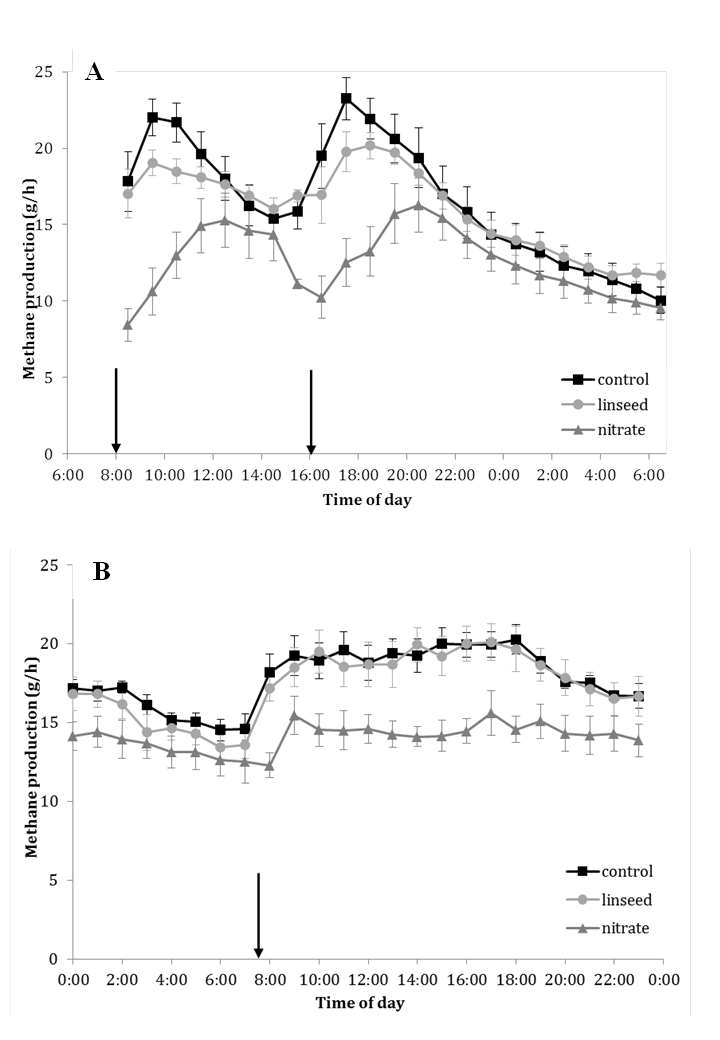

Supplement: S3 Fig — Error bars indicate SE. Arrows indicate time of feeding. (TIF) [file pone.0140282.s003.tif]

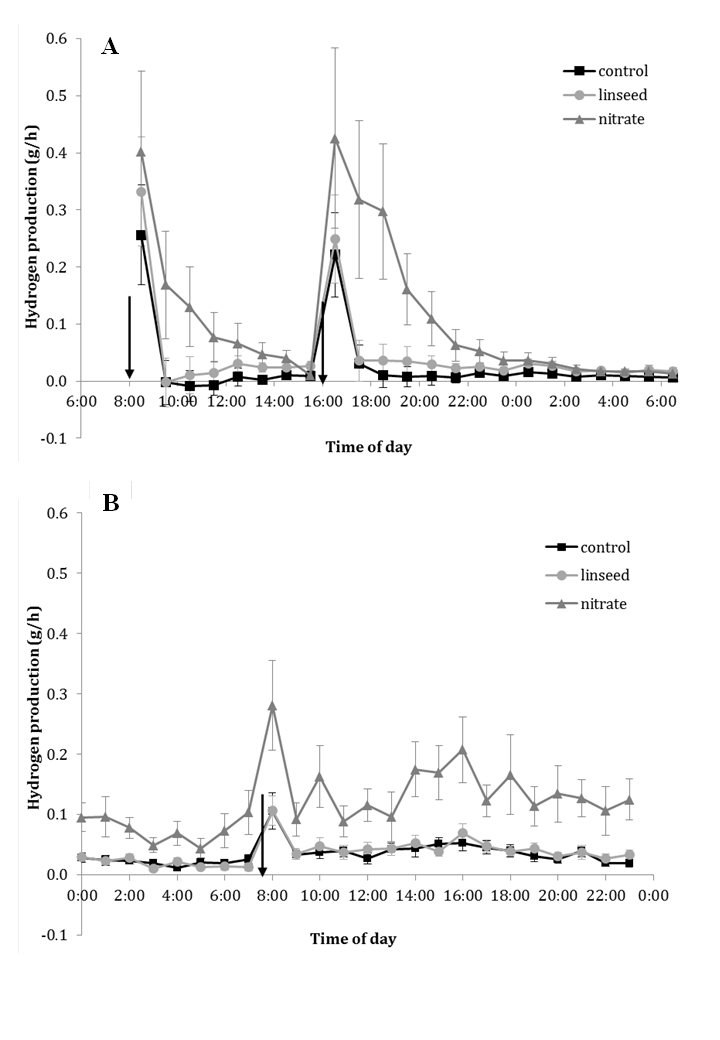

Supplement: S4 Fig — Error bars indicate SE. Arrows indicate time of feeding. (TIF) [file pone.0140282.s004.tif]
