# Supplementary material for: Does Dietary Mitigation of Enteric Methane Production Affect Rumen Function and Animal Productivity in Dairy Cows?
Source: PLoS One. 2015 Oct 28;10(10):e0140282. doi: 10.1371/journal.pone.0140282 (PMC4624802; doi:10.1371/journal.pone.0140282)
Supplement: S1 Table — Fw, forward primer; NGS, next generation sequencing; qPCR, quantative PCR; Rev, reverse primer; Ta, annealing temperature. (DOCX) [file pone.0140282.s005.docx]

**S1 Table.** **Targeted primers used for quantification of bacteria, archaea, fungi and protozoa by qPCR and NGS.**

| Target |  | Sequence 5’-… - 3’ | T_a_ | reference |
| --- | --- | --- | --- | --- |
| **qPCR** |  |  |  |  |
| Bacteria, | Fw^2^ | GTG STG CAY GGY TGT CGT CA | 61°C | Maeda *et al*.  [[1](#_ENREF_1)] |
| 16S | Rev^3^ | ACG TCR TCC MCA CCT TCC TC |  |  |
| Archaea, | Fw | TTC GGT GGA TCD CAR AGR GC | 56°C | Denman *et al*. |
| mcrA | Rev | GBA RGT CGW AWC CGT AGA ATC C |  | [[2](#_ENREF_2)] |
| Fungi, | Fw | GAG GAA GTA AAA GTC GTA ACA AGG TTT C | 62°C | Denman and McSweeney [[3](#_ENREF_3)] |
| 18S/ITS | Rev | CAA ATT CAC AAA GGG TAG GAT GAT T |  |  |
| Protozoa, | Fw | GCT TTC GWT GGT AGT GTA TT | 55°C | Sylvester *et al*. |
| 18S | Rev | CTT GCC CTC YAA TCG TWC T |  | [[4](#_ENREF_4)] |
| **NGS** |  |  |  |  |
| Bacteria, | Fw | AGA GTT TGA TCM TGG CTC AG | 58°C | Li *et al.* |
| 16S | Rev | CTG CTG CCT YCC GTA |  | [[5](#_ENREF_5)] |
| Archaea, | Fw | GCT CAG TAA CAC GTG G | 65°C | DeLong *et al.* [[6](#_ENREF_6)] |
| 16S | Rev | TTA CCG CGG CKG CTG |  | Zhou *et al.* [[7](#_ENREF_7)] |

Fw, forward primer; NGS, next generation sequencing; qPCR, quantative PCR; Rev, reverse primer; T_a_, annealing temperature.

**S1 Table References**

1. Maeda H, Fujimoto C, Haruki Y, Maeda T, Kokeguchi S, Petelin M, et al. Quantitative real-time PCR using TaqMan and SYBR Green for Actinobacillus actinomycetemcomitans, Porphyromonas gingivalis, Prevotella intermedia, tetQ gene and total bacteria. FEMS Immunology & Medical Microbiology. 2003;39(1):81-6.

2. Chilliard Y, Glasser F, Ferlay A, Bernard L, Rouel J, Doreau M. Diet, rumen biohydrogenation and nutritional quality of cow and goat milk fat. European Journal of Lipid Science and Technology. 2007;109(8):828-55.

3. Denman SE, McSweeney CS. Development of a real-time PCR assay for monitoring anaerobic fungal and cellulolytic bacterial populations within the rumen. FEMS Microbiology Ecology. 2006;58(3):572-82.

4. Sylvester JT, Karnati SKR, Yu Z, Morrison M, Firkins JL. Development of an Assay to Quantify Rumen Ciliate Protozoal Biomass in Cows Using Real-Time PCR. The Journal of Nutrition. 2004 December 1, 2004;134(12):3378-84.

5. Li H, Zhang Y, Li D-s, Xu H, Chen G-x, Zhang C-g. Comparisons of different hypervariable regions of rrs genes for fingerprinting of microbial communities in paddy soils. Soil Biology and Biochemistry. 2009;41(5):954-68.

6. DeLong EF. Archaea in coastal marine environments. Proceedings of the National Academy of Sciences. 1992 June 15, 1992;89(12):5685-9.

7. Zhou M, Hernandez-Sanabria E, Guan LL. Characterization of Variation in Rumen Methanogenic Communities under Different Dietary and Host Feed Efficiency Conditions, as Determined by PCR-Denaturing Gradient Gel Electrophoresis Analysis. Applied and Environmental Microbiology. 2010 June 15, 2010;76(12):3776-86.
